# Homotypic fusion of endoplasmic reticulum membranes in plant cells

**DOI:** 10.3389/fpls.2013.00514

**Published:** 2013-12-18

**Authors:** Miao Zhang, Junjie Hu

**Affiliations:** ^1^Tianjin Key Laboratory of Protein Science and Department of Genetics and Cell Biology, College of Life Sciences, Nankai UniversityTianjin, China; ^2^National Laboratory of Biomacromolecules, Institute of Biophysics, Chinese Academy of SciencesBeijing, China

**Keywords:** endoplasmic reticulum, membrane proteins, membrane fusion, GTPase, plant development

## Abstract

The endoplasmic reticulum (ER) is a membrane-bounded organelle whose membrane comprises a network of tubules and sheets. The formation of these characteristic shapes and maintenance of their continuity through homotypic membrane fusion appears to be critical for the proper functioning of the ER. The atlastins (ATLs), a family of ER-localized dynamin-like GTPases, have been identified as fusogens of the ER membranes in metazoans. Mutations of the ATL proteins in mammalian cells cause morphological defects in the ER, and purified Drosophila ATL mediates membrane fusion *in vitro*. Plant cells do not possess ATL, but a family of similar GTPases, named root hair defective 3 (RHD3), are likely the functional orthologs of ATLs. In this review, we summarize recent advances in our understanding of how RHD3 proteins play a role in homotypic ER fusion. We also discuss the possible physiological significance of forming a tubular ER network in plant cells.

## INTRODUCTION

The endoplasmic reticulum (ER) is the origin of the endomembrane system in eukaryotic cells. Secretory proteins and most of the integral membrane proteins are synthesized and folded by the ER, cellular membrane sources are generated on the ER, and calcium ions are stored in the lumen of the ER. Morphologically, the ER membranes can adopt a tubular shape or form flattened cisternal structures, called ER sheets (Shibata et al., 2006). Despite the ER representing one of the largest intracellular membrane surfaces, the ER membranes in each cell are continuous as one entity. Though the sheets may be stacked by helicoidal membrane motifs (Terasaki et al., 2013), tubules often extend from sheets and are connected via three-way junctions into a reticular network (Lee and Chen, 1988; Hu et al., 2008). In some areas of the ER, tubules and sheets are interspersed in fenestrated structures (West et al., 2011).

The formation and maintenance of a continuous membrane system requires constant fusion of identical membranes. A similar fusion process includes the merger of mitochondrial membranes, in which dynamin-like GTPases mitofusin/Fzo1 and OPA1/Mgm1 play important roles (Chan, 2006; Hoppins et al., 2007). However, how such homotypic fusion occurs is poorly understood. In contrast, the merger of heterotypic membranes, such as the fusion of viral and cellular membranes or transport vesicles with target membranes, has been studied intensively. In viral fusion, the membranes are pulled together by an intramolecular conformational change in a single protein (Harrison, 2008). In vesicular fusion, three t-SNARE proteins in one membrane and a v-SNARE partner in the other zipper up to form a four-helix bundle in the fused lipid bilayer (Jahn and Scheller, 2006; Martens and McMahon, 2008; Wickner and Schekman, 2008; Sudhof and Rothman, 2009).

In mammalian cells, defects in branch formation of the ER network, a sign of a lack of sufficient homotypic fusion, was recently linked to a class of membrane-bound, dynamin-like GTPases named atlastins (ATLs; Rismanchi et al., 2008; Hu et al., 2009). Lipid bilayer fusion can be achieved with purified Drosophila ATL (Orso et al., 2009; Bian et al., 2011). Following the discovery of ATLs, Sey1p in yeast cells was identified as a functional ortholog (Anwar et al., 2012). The deletion of Sey1p drastically delays ER fusion during mating, and the re-introduction of Sey1p restores the defects (Anwar et al., 2012). Similar to ATL, reconstituted Sey1p is capable of fusing vesicles *in vitro* (Anwar et al., 2012).

Plant cells do not possess ATL homologs; however, a GTPase called root hair defective 3 (RHD3) is related to Sey1p in regards to sequence (Brands and Ho, 2002) and has been suggested to mediate the fusion of ER membranes (Hu et al., 2009; Chen et al., 2011). Although the mechanisms of ER fusion may be conserved in plant cells, the plant ER exhibits several unique features: a prominent cortical ER (West et al., 2011); participation in plasmodesma formation, a specialized intercellular ER connection (Gupton et al., 2006); and the movement of Golgi bodies along ER tubules (Boevink et al., 1998; Faso et al., 2009; Sparkes et al., 2009). These characteristics imply that homotypic ER fusion in plant cells may play distinct roles.

## HOMOTYPIC ER FUSION IN MAMMALIAN AND YEAST CELLS

**FIGURE 1:**
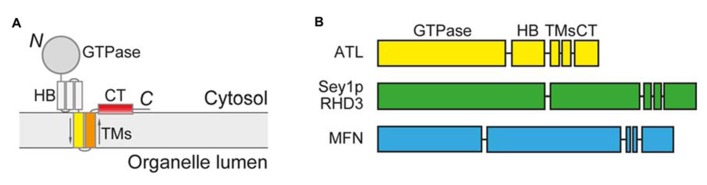
**Membrane topology and domain structure of ATL-related GTPases.** **(A)** Membrane topology of ATL-related GTPases. 3HB, three-helix bundle; TMs, transmembrane segments; CT, C-terminal tail. **(B)** Domain structure of ATL-related GTPases.

The first clue of homotypic fusion of the ER membranes in mammalian cells came from overexpression of mutant forms of ATL, a membrane-bound GTPase (Rismanchi et al., 2008). ATL mutations cause unbranched ER morphology, indicating a lack of fusion between ER tubules. ATL is anchored in the membrane by two closely spaced transmembrane (TM) segments, exposing both the N-terminal GTPase domain and the C-terminal tail (CT) to the cytosol (Figure **1A**). ATL localizes mostly in the tubular region of the ER and interacts with the ER tubule resident proteins reticulons and DP1/Yop1p (Hu et al., 2009), two families of integral membranes that induce high curvature in the ER membranes to form tubules (Voeltz et al., 2006; Hu et al., 2009). ATL belongs to the dynamin superfamily of GTPases. A related family member, mitofusin (MFN), shares membrane topology and domain structures with ATL (Figure **1B**) and is known to mediate fusion of the outer mitochondrial membranes (Hermann et al., 1998; Chen et al., 2003). Thus, ATL is likely responsible for fusion of the ER membranes. In fact, depletion of ATL causes unbranched ER in mammalian cells (Hu et al., 2009), and ER fragmentation in *Drosophila* (Orso et al., 2009). In addition, antibodies against ATL block ER network formation *in vitro* in Xenopus egg membrane extracts (Hu et al., 2009). Most convincingly, purified *Drosophila* ATL is able to mediate vesicle fusion *in vitro* when reconstituted into proteoliposomes (Orso et al., 2009).

Atlastins is conserved in most metazoa, but it is not found in many organisms in which the tubular ER network is properly formed. *Saccharomyces cerevisiae* is among these organisms, but a similar GTPase named Sey1p was recently identified as an ER fusogen (Anwar et al., 2012). Sey1p is a synthetic enhancer for Yop1p (Brands and Ho, 2002), one of the ER tubule shaping proteins, and it not so surprisingly plays a role in tubular ER network formation. Cells lacking Sey1p exhibit minor morphological defects in the ER (Hu et al., 2009), but an ER SNARE Ufe1p is required when SEY1 is deleted and may represent an alternative ER fusogen in yeast (Anwar et al., 2012).

## ASSAYS FOR HOMOTYPIC ER FUSION

Several assays have been developed or adapted to verify candidates for homotypic ER fusion. The first assay visualizes the integrity of the ER in yeast cells (Hu et al., 2009). Deletion of Sey1p and either Rtn1p or Yop1p, membrane proteins that shape the ER tubules, causes drastic ER defects; in particular, the tubular ER network is mostly converted to sheets and the large areas of the cortex are void of ER, indicating a lack of ER fusion. When wild-type Sey1p is re-introduced, fusion activities in the ER resume and ER morphology, visualized by GFP-labeled ER-resident protein Sec63p, restored. The rescue of the ER defects in *sey1*Δ *rtn1*Δ or *sey1*Δ *yop1*Δ cells by certain proteins indirectly indicates its ability to mediate ER fusion.

The second assay monitors ER fusion during the mating of yeast cells (Anwar et al., 2012). Similar assays have been used to study nuclear fusion or mitochondrial fusion. Haploid yeast cells expressing cytosolic GFP remated with cells expressing a red fluorescent protein (RFP)-containing ER marker (ss-RFP-HDEL). When cell fusion occurs between two types of cells, the cytosolic GFP of one cell rapidly diffuses to the other cell, marking the starting point for ER fusion. The efficiency of ER fusion is monitored by the equilibration of the RFP signal between two cells. Cells lacking Sey1p exhibit a significant delay in ER fusion, but plasmid-driven expression of Sey1p would restore such a defect. To test whether certain molecules mediate ER fusion *in vivo*, they are expressed in haploid cells with either cytosolic or ER marker, both of which lack Sey1p. ER fusion is then measured and compared to that of untransformed *sey1*Δ cells.

Finally, *in vitro* fusion assays have been adapted from studies of SNARE-mediated fusion (Weber et al., 1998; Scott et al., 2003). For lipid mixing tests, full-length fusion candidates are purified in detergent and reconstituted into proteoliposomes upon detergent removal. A group of vesicles incorporates lipids labeled with two fluorophores (NBD and rhodamine) at quenching concentrations. When these vesicles fuse with vesicles containing unlabeled lipids, the labeled lipids are diluted and subsequently dequenched. The increase in fluorescence correlates with the level of lipid mixing resulting from fusion. To further distinguish hemi-fusion and full fusion, two fluorescent dyes are incorporated as a FRET pair into reconstituted vesicles and the FRET signal measured as an indicator of the content mixing resulting from full fusion of the two bilayers (Zucchi and Zick, 2011).

Combining these three assays, ATL and Sey1p proteins were tested and confirmed to mediate fusion of the ER membranes (Table **1**). Based on the results for *Drosophila* ATL, the fusion reaction can lead to efficient content mixing with nearly no lysis of the membranes (Liu et al., 2012). Using the same criteria, RHD3 has recently joined the list of ER fusogens as a plant ortholog of ATL and Sey1p (Zhang et al., 2013).

**Table 1:** Assays for homotypic ER fusion.

GTPases	ER morphology in yeast cells	In cell fusion in yeast cells	Lipid mixing *in vitro*	Content mixing *in vitro*
ATL	✓Anwar et al. (2012)	✓Anwar et al. (2012)	✓Orso et al. (2009)	✓Liu et al. (2012)
Sey1p	✓Hu et al. (2009)	✓Anwar et al. (2012)	✓Anwar et al. (2012)	ND
RHD3	✗Chen et al. (2011), ✔Zhang etal. (2013)	✓Zhang et al. (2013)	✓Zhang et al. (2013)	ND

*✓, Functional; ✗, Non-functional; ND, not determined.*

## RHD3 FAMILY PROTEINS AS PLANT ER FUSOGENS

The components involved in shaping ER tubules are conserved among eukaryotes. Shortly after reticulons and DP1/Yop1p were found in mammals and yeast cells (Voeltz et al., 2006), the plant orthologs were analyzed and confirmed to have the same role (Nziengui et al., 2007; Sparkes et al., 2010). Similarly, when ATL and Sey1p were shown to mediate ER fusion (Hu et al., 2009), a related protein family, RHD3, became very plausible candidates for ER fusogens in plant cells.

RHD3 was initially discovered by a genetic screen of root hair development (Schiefelbein and Somerville, 1990). Mutations in RHD3 proteins cause short and wavy root hairs and a dwarf phenotype (Schiefelbein and Somerville, 1990; Wang et al., 1997). A role for RHD3 in ER morphogenesis was indicated, even before the characterization of ATL and Sey1p, when *rhd3-1* plants (A575V) were found to contain “cable-like” ER (Zheng et al., 2004), a defect reminiscent of ATL mutations or depletion in mammalian cells. Subsequently, several other RHD3 point mutants or null mutants were found to result in the same ER defects (Zheng et al., 2004; Chen et al., 2011; Stefano et al., 2012; Zhang et al., 2013), supporting the notion that RHD3 plays a role in connecting ER tubules. In addition to RHD3, two RHD3-like proteins were found in *Arabidopsis* (Hu et al., 2003). RL1 is expressed only in pollen, whereas RL2 is expressed ubiquitously, but both are present at very low levels. Individual deletions of the RL proteins show no detectable defects in plant development. However, over-expression of RL2 rescues the *rhd3-1* mutant (Chen et al., 2011), suggesting a redundant role among these proteins.

Similar to ATL and Sey1p, RHD3 localizes mainly to the tubular ER network; colocalizes with HVA22 (Chen et al., 2011), a plant ortholog of DP1/Yop1p; and its homolog RL2 interacts with plant reticulons (Lee et al., 2013). However, RHD3 and Sey1p are not thought to be interchangeable (Chen et al., 2011), i.e. Sey1p cannot rescue the rhd3 mutant, and RHD3 cannot replace Sey1p in yeast. To test the possibility that RHD3 and Sey1p act differently, the yeast complementation assay was recently revisited. Either RHD3 or the RL proteins was expressed under the control of the endogenous SEY1 promoter in *sey1*Δ *yop1*Δ cells, and the results indicated that RHD3 family members are capable of restoring ER defects in *sey1*Δ *yop1*Δ cells (Zhang et al., 2013). Though Sey1p might not be functional in the setting of plant cells, these findings suggest that RHD3 and Sey1p act similarly in yeast cells and *in vitro* as purified proteins. Using the same assays that are applicable to ATL and Sey1p, the RHD3 proteins fuse the ER in cells and lipid membranes *in vitro*, confirming that they are ER fusogens in plant cells(Zhang et al., 2013).

Some fusion events have been observed between peripheral ER tubules in plant cells lacking RHD3 (Stefano et al., 2012). As neither RL is dispensable on the rhd3-null background (Stefano et al., 2012), these fusion activities are likely carried out by RL proteins, even though their levels are very low compared to RHD3. The strong ER-branching defect in *rhd3* mutants suggests that RHD3 is the major force to connect ER membranes, but whether another ER fusogen exists in plant cells remains to be tested.

## MECHANISMS FOR HOMOTYPIC ER FUSION IN PLANT CELLS

Given the domain structure and functional similarity among ATL, Sey1p, and RHD3, these GTPases likely utilize conserved mechanisms to mediate fusion. How RHD3 performs the fusion reaction in plant cells is not clear, but significant progress has been made in understanding ATL-mediated homotypic fusion. Crystal structures of the N-terminal cytosolic domain of human ATL1 have been determined (Bian et al., 2011; Byrnes and Sondermann, 2011), revealing a GTPase domain and three-helix bundle (3HB) connected by a linker region. The GTPase domains face each other to form a nucleotide-dependent dimer in all structures, but the 3HBs are positioned differently. In the structure obtained when only GDP is added, the 3HBs following the GTPase domains point in opposite directions; in another structure in which GDP and phosphate are present, the 3HBs are parallel to one another and crossover to dock against the GTPase domain of the partner molecule; and in the recent structures in which GMPPNP, a non-hydrolysable analog of GTP, or GDP and aluminum fluoride were used, the 3HBs come even closer in the crossed over conformation (Byrnes et al., 2013). Taken together, the evidence indicates that GTP binding induces interactions between ATL molecules across the apposing membranes, and subsequent GTP hydrolysis causes conformational changes through steps that are not entirely clear to force the 3HBs of engaging ATL molecules come very close, pulling the two membranes together.

In addition to the GTP-dependent mechanism, the CT of ATL forms an amphipathic helix that binds and perturbs the membrane bilayer, facilitating the fusion process, and the TM segments are required for efficient fusion, probably by mediating nucleotide-independent oligomerization of ATL molecules (Liu et al., 2012).

The homotypic interactions of RHD3 protein have been confirmed (Chen et al., 2011), but the mechanisms of RHD3-mediated membrane fusion remain to be tested. Notably, the region between the GTPase domain of RHD3 and the TM segments is much longer than that of the 3HB in ATL. Based on secondary structure prediction, this region likely forms a helical bundle, but whether it binds to the GTPase domain, or even in a similar manner as ATL, is largely unknown.

## PERSPECTIVE

Homotypic ER fusion appears to be a conserved process among eukaryotic cells. In plants, the process is mediated primarily by the RHD3 family of proteins. Like the ATL family of proteins, members of the RHD3 family are present ubiquitously. However, the prominent defects caused by mutations in RHD3 occur in cells with long protrusions, namely the root hairs, which is reminiscent of ATL1 with cortical spinal motor neurons. Though complete deletion of ATLs in mammals is yet to be achieved, the loss of RHD3 and either of the RL proteins results in lethality (Zhang et al., 2013). These results suggest that, at least in plants, more cell types require the presence of the RHD3 family than is previously thought.

One important question that remains to be addressed is the role of the RHD3 family *in vivo*. For example, there may be a link between these GTPases and the ER-plasma membrane (PM) contact sites. The non-branched or “cable-like” ER is often clustered in the cell body rather than present in the cortex. It is reasonable to speculate that RHD3 mutations perturb the general functions of the ER-PM contact sites, such as calcium signaling or lipid sensing and transfer. The formation of plasmodesmata also relies on coordination of the PM and cortical ER, and ER tubules from neighboring cells need to be fused in the nanopores. Will plasmodesmata be properly generated in RHD3 mutants? If not, what happens to the nanopores? Finally, Golgi body distribution and movement along the tubular ER network has been shown to be affected in rhd3-1 (Chen et al., 2011) or gom8 (Stefano et al., 2012), an EMS mutant of RHD3 (P701S). Does this defect directly lead to impaired plant development? The hope is that answering these questions will have an impact on our understanding of the correlation between the morphology of organelles and their functions.

## Conflict of Interest Statement

The authors declare that the research was conducted in the absence of any commercial or financial relationships that could be construed as a potential conflict of interest.
